# Effects of standardized ileal digestible lysine:crude protein ratio and the use of non-protein nitrogen on growth performance of 11- to 25-kg pigs

**DOI:** 10.1093/jas/skag013

**Published:** 2026-01-20

**Authors:** Jessica L Smallfield, Mike D Tokach, Katelyn N Gaffield, Robert D Goodband, Jason C Woodworth, Joel M Derouchey, Jordan T Gebhardt, Keith D Haydon, Alan J Warner, Chad W Hastad, Dwight J Shawk, Noah C Gainey, Henrique S Cemin, Jose A Soto

**Affiliations:** Department of Animal Sciences and Industry, College of Agriculture, Kansas State University, Manhattan, KS 66506-0201, United States; Department of Animal Sciences and Industry, College of Agriculture, Kansas State University, Manhattan, KS 66506-0201, United States; Department of Animal Sciences and Industry, College of Agriculture, Kansas State University, Manhattan, KS 66506-0201, United States; Department of Animal Sciences and Industry, College of Agriculture, Kansas State University, Manhattan, KS 66506-0201, United States; Department of Animal Sciences and Industry, College of Agriculture, Kansas State University, Manhattan, KS 66506-0201, United States; Department of Animal Sciences and Industry, College of Agriculture, Kansas State University, Manhattan, KS 66506-0201, United States; Department of Diagnostic Medicine/Pathobiology, College of Veterinary Medicine, Kansas State University, Manhattan, KS 66506-0201, United States; CJ America, Downers Grove, IL, 60515, United States; New Fashion Pork, Jackson, MN, 56143, United States; New Fashion Pork, Jackson, MN, 56143, United States; Hord Family Farms, Bucyrus, OH, 44820, United States; Hord Family Farms, Bucyrus, OH, 44820, United States; Hubbard Feeds, Mankato, MN, 56001, United States; Alltech, Nicholasville, KY, 40356, United States

**Keywords:** amino acids, crude protein, lysine, non-protein nitrogen, nursery pigs

## Abstract

Three experiments were conducted to determine if nitrogen is a limiting factor for growth performance when feeding low protein, amino acid (AA) fortified diets and determine the effects of standardized ileal digestible lysine to crude protein (SID Lys:CP) ratio on growth performance of 11- to 25-kg pigs. In Exp. 1,981 pigs ([Fast Large White × PIC L02] × PIC 800; initially 10.3 ± 0.19 kg) were used in a 21-d study. Diets were corn-soybean meal-based consisting of: 1) a low level of feed-grade AA with a SID Lys:CP ratio of 6.0%; 2) a moderate level of feed-grade AA with a SID Lys:CP ratio of 6.5%; 3) a high level of feed-grade AA with a SID Lys:CP ratio of 7.0%; 4) diet 3 with added diammonium phosphate (DAP) added to achieve a SID Lys:CP ratio of 6.5%; and 5) diet 3 with L-Gly added to achieve a SID Lys:CP ratio of 6.5%. Average daily gain (ADG) was unaffected by dietary treatment but gain:feed ratio (G:F) decreased (linear, *P *= 0.002; quadratic, *P *= 0.054) as SID Lys:CP ratio exceeded 6.5%. Adding DAP or L-Gly to the high feed-grade AA diet increased (*P *≤ 0.003) G:F compared to pigs fed the high feed-grade AA diet. In Exp. 2, 4,167 pigs (337 × 1050, PIC; initially 13.0 ± 0.27 kg) were used in a 14-d study. Diets were corn-soybean meal-based, and treatments arranged in a 2 × 5 factorial with main effects of SID Lys (1.15 or 1.30%) and SID Lys:CP ratio (6.00, 6.22, 6.46, 6.72, and 7.00%). Overall ADG was unaffected by dietary treatment; however, a SID Lys:CP × SID Lys interaction was observed for G:F (linear, *P *= 0.012) where increasing SID Lys:CP ratio decreased (linear, *P *< 0.001) G:F at both SID Lys levels with a more pronounced effect in diets formulated to 1.15% SID Lys. Lastly, Exp. 3 used 5,059 pigs (PIC 800 × Camborough and DNA 600 × 241; initially 11.0 ± 0.90 kg) in an 18-d trial. Treatment diets were arranged in a 2 × 6 factorial with main effects of dried distillers grains with solubles (DDGS; 0 or 15%) and SID Lys:CP ratio (6.01, 6.22, 6.45, 6.70, 6.97, and 7.26%). Overall, ADG was unaffected by dietary treatment, but a SID Lys:CP × DDGS interaction was observed (linear, *P *< 0.001) where G:F increased then decreased (quadratic, *P *< 0.001) in diets without DDGS, whereas in the diets with DDGS, G:F decreased (quadratic, *P *≤ 0.002) as SID Lys:CP ratio increased above 6.45%. In summary, a SID Lys:CP ratio greater than approximately 6.5% decreased G:F, but adding a protein or non-protein nitrogen source to low protein diets formulated above this ratio improves G:F.

## Teaser

Feeding a standardized ileal digestible lysine:crude protein ratio greater than 6.5% negatively affects feed efficiency in 11- to 25-kg pigs.

## Introduction

Although crude protein (CP) is a fundamental component of swine diets, the primary nutritional focus is supplying adequate essential amino acids (EAA) and sufficient nitrogen (N) to support the endogenous synthesis of non-essential amino acids (NEAA). Because Lys is the first limiting amino acid (AA) in corn-soybean meal-based swine diets, it is present in the lowest amount relative to the pig’s requirement. Therefore, the requirements for other EAA can be expressed relative to Lys (Boisen et al. 2000). Hence, an ideal protein profile can be used to formulate diets that meet the pig’s requirements with minimal excess (Zhao et al. 2019; Yang et al. 2020). This concept has been expanded to include an optimal balance between EAA and NEAA (Wang and Fuller, 1989; Lenis et al. 1999). As the dietary CP level decreases, the supply of N available for endogenous NEAA synthesis also declines. Therefore, when formulating low protein, feed-grade AA fortified diets, a NEAA nitrogen (NEAA-N) source, or a requirement for total N should be considered (Boisen 1997).

The NRC (2012) estimates a standardized ileal digestible (SID) Lys requirement of 1.23% and total dietary N of 3.02% for pigs weighing 11 to 25 kg. These values imply that diets should be formulated to maintain a SID Lys:CP ratio at or below 6.5% for 11- to 25-kg pigs. Maintaining the ratio below this threshold ensures that the diet contains sufficient intact protein to supply N for endogenous NEAA synthesis. However, excess CP in the diet may lead to excess AA which must be deaminated and excreted in urine (Van Milgen and Dourmad 2015; Wang et al. 2018). Conversely, if the SID Lys:CP ratio is too high, the diet lacks sufficient N for the synthesis of NEAA (Gloaguen et al. 2014).

The use of feed-grade AA in low protein diets has greatly benefited the swine industry by lowering feed costs, reducing N excretion, and minimizing swine production’s environmental impact. Diets high in CP can increase the amount of undigested proteins in the large intestine, which may promote the proliferation of pathogenic bacteria and cause digestive issues (Heo et al. 2009). As a result, partially replacing intact protein sources, like soybean meal (SBM), with feed-grade AA can help maintain or even enhance pig performance, particularly feed efficiency (Gloaguen et al. 2014). However, Gotterbarm et al. (1998) and Heger et al. (1998) observed that growth performance may be decreased due to low dietary CP despite meeting EAA requirements. This is thought to be because of decreasing NEAA-N to total N ratio. When dietary NEAA-N is insufficient, the required N may come from the catabolism of EAA, reducing the efficiency of EAA utilization for lean growth (Buchinski et al. 2024a). If the dietary CP is inadequate, a N source may be provided in the diet to provide sufficient N for the synthesis of NEAA. This concept is supported by research conducted by Mansilla et al. (2017) and Buchinski et al. (2024b), who observed that ammonia can serve as a non-protein nitrogen (NPN) source when N is limiting in diets to recover this loss in growth performance.

Although a SID Lys:CP ratio of 6.5% for 11- to 25-kg pigs can be implied from the NRC (2012), further research is needed to validate this ratio. Therefore, the objectives of these three experiments utilizing 11- to 25-kg pigs were 1) to determine whether N is a limiting factor on growth performance when feeding low protein, feed-grade AA fortified diets, and 2) to determine the optimal SID Lys:CP ratio in diets containing different inclusions of SBM. It was hypothesized that under-feeding N would negatively affect growth performance and that an optimum balance between Lys and CP is necessary when feeding low protein, AA fortified diets.

## Materials and methods

The Kansas State University Institutional Animal Care and Use Committee approved the protocols used in these experiments (IACUC #4892, #5008, #4996). All experiments were conducted at commercial research sites, and pigs were provided *ad libitum* access to feed and water. Experiment 1 was conducted in southwest Minnesota (New Fashion Pork, Round Lake, MN). The facility contained 52 pens and was completely enclosed, environmentally controlled, and mechanically ventilated. Each pen contained a three-hole, dry self-feeder and a bowl waterer. Experiment 2 was conducted in north central Ohio (Hord Family Farms, Bucyrus, OH). A total of 160 pens were used with 80 double-sided, five-hole stainless steel fence-line feeders, each feeding two adjacent pens. Each pen was also equipped with a cup waterer. Experiment 3 was conducted in southwest Minnesota (Hubbard Feeds, Sleepy Eye, MN). The barns were mechanically ventilated with totally slatted floors. A total of 143 pens were used, each equipped with a five-hole stainless steel dry self-feeder and a bowl waterer.

In each experiment, corn, SBM, and dried distillers grains with solubles (DDGS; Exp. 3) samples were collected and analyzed for AA and proximate analysis. Analyzed nutrient values were then used in diet formulation using NRC (2012) SID coefficients. Net energy of SBM was set at 90% that of corn NE (Stein, 2024). As a result, NE values and the SID Lys:NE ratios of experimental diets varied slightly.

### Experiment 1

#### Animals and treatment structure

The objective of Exp. 1 was to determine if N is a limiting factor to maintain feed efficiency when feeding low protein, feed-grade AA fortified diets. A total of 981 pigs ([Fast Large White × PIC L02] × PIC 800; initially 10.3 ± 0.19 kg and approximately 18 days after weaning) were used in a 21-d growth study. Pigs were housed in mixed-sex pens and pens were allotted to one of five dietary treatments in a randomized complete block design with initial weight as a blocking factor. There were 19 to 20 pigs per pen and 10 pens per treatment. Prior to phase 3 diet formulation, composite corn and SBM samples were submitted (Market 1 AgState, Cherokee, IA) for proximate analysis and AA profile using NIR (Table 1). Treatment diets were corn-SBM-based and consisted of: 1) a low level of feed-grade AA with a SID Lys:CP ratio of 6.0%; 2) a moderate level of feed-grade AA with a SID Lys:CP ratio of 6.5%; 3) a high level of feed-grade AA with a SID Lys:CP ratio of 7.0%; 4) diet 3 with added diammonium phosphate (DAP) to achieve a SID Lys:CP ratio of 6.5%; and 5) diet 3 with added L-Gly to achieve a SID Lys:CP ratio of 6.5% (Table 2). DAP was used as an inorganic N source while L-Gly was used as a NEAA source to determine if differences in N source affected performance. Feed-grade AA replaced SBM in the diets to form the first three dietary treatments. Dietary additions of feed-grade AA were adjusted to meet or exceed NRC (2012) AA requirement estimates in relation to Lys for Met and Cys, Thr, Trp, Val, Ile, and His. Treatment diets were manufactured at the New Fashion Pork feed mill (Round Lake, MN). Titanium dioxide was included at 0.4% in the diets as an indigestible marker to determine apparent total tract digestibility (ATTD) of dry matter (DM) and CP. Diet samples were analyzed at the University of Missouri Agricultural Experiment Station Chemical Laboratory for CP (Method 990.03; AOAC International 2006), ash, DM (Method 935.29; AOAC International 2006), ether extract (ANKOM Technology 2004), crude fiber (ANKOM Technology 2005), and complete AA profile (Method 982.30; AOAC International 2006; Table 3).

**Table 1:** Chemical composition of soybean meal (SBM) and corn for Exp. 1 (as-fed basis).^1^

Nutrient, %	SBM	Corn
** CP**	46.09	7.23
** Dry matter**	86.97	84.77
** Crude fat**	0.58	3.60
** Crude fiber**	3.86	1.77
** Ash**	5.77	1.10
** Amino acids**		
** Arg**	3.14	0.30
** Cys**	0.57	0.15
** His**	1.15	0.19
** Ile**	1.97	0.23
** Leu**	3.32	0.88
** Lys**	2.75	0.23
** Met**	0.57	0.14
** Phe**	2.17	0.27
** Thr**	1.77	0.25
** Trp**	0.63	0.05
** Val**	2.10	0.32

1 Samples were analyzed for proximate analysis and AA profile using NIR (Market 1 AgState, Cherokee, IA). These values, using NRC (2012) standardized ileal digestibility coefficients, were then used in diet formulation.

**Table 2:** Diet composition, Exp. 1 (as-fed basis).^1^

Feed-grade AA:	Low	Moderate	High	High	High
Added N:	None	None	None	DAP^2^	Glycine
SID Lys:CP:	6.0:1	6.5:1	7.0:1	6.5:1	6.5:1
**Ingredient, %**					
** Corn**	62.12	66.78	70.10	69.52	68.97
** Soybean meal**	33.75	28.55	24.74	24.74	24.74
** Calcium carbonate**	0.68	0.70	0.70	1.20	0.70
** Monocalcium P (21% P)**	0.95	1.00	1.05	—	1.05
** Salt**	0.60	0.60	0.61	0.61	0.61
** L-Lys-HCl**	0.40	0.55	0.66	0.66	0.66
** DL-Met**	0.25	0.29	0.32	0.32	0.32
** L-Trp**	0.04	0.06	0.09	0.09	0.09
** L-Val**	0.13	0.21	0.28	0.28	0.28
** L-Ile**	—	0.06	0.13	0.13	0.13
** Thr^3^**	0.27	0.35	0.42	0.42	0.42
** L-His-HCl**	—	0.03	0.08	0.08	0.08
** Vitamin premix^4^ ^,^ ^5^**	0.25	0.25	0.25	0.25	0.25
** Trace mineral premix^6^**	0.15	0.15	0.15	0.15	0.15
** Glycine**	—	—	—	—	1.14
** Diammonium phosphate (18% N)**	—	—	—	1.14	—
** Titanium dioxide**	0.40	0.40	0.40	0.40	0.40
** Copper chloride^7^**	0.03	0.03	0.03	0.03	0.03
**Total**	100	100	100	100	100
**Calculated analysis**					
** SID AA**					
** Lys, %**	1.25	1.25	1.25	1.25	1.25
** Ile:Lys**	57	55	55	55	55
** Leu:Lys**	117	108	101	100	100
** Met:Lys**	39	40	42	42	42
** Met and Cys:Lys**	58	58	58	58	58
** Thr:Lys**	67	67	67	67	67
** Trp:Lys**	20.3	20.0	20.3	20.3	20.3
** Val:Lys**	72	72	72	72	72
** His:Lys**	36	34	34	34	34
** Arg:Lys**	93	81	73	73	73
** Phe and Tyr:Lys**	111	99	90	90	90
** Total Lys, %**	1.39	1.38	1.37	1.37	1.37
** SID EAA:NEAA**	0.84	0.89	0.94	0.94	0.83
** NE, kcal/kg**	2,509	2,525	2,537	2,521	2,530
** SID Lys:NE, g/Mcal**	4.98	4.95	4.93	4.95	4.93
** CP, %**	20.9	19.2	17.9	19.2	19.2
** Ca, %**	0.63	0.63	0.63	0.63	0.63
** STTD P, %**	0.47	0.47	0.47	0.47	0.47
** Ca:P**	1.03	1.06	1.07	1.08	1.07

1 Diets were fed from 10.3 to 22.0 kg.

2 Diammonium phosphate.

3 Thr Pro 80%; CJ America-Bio, Downers Grove, IL.

4 Provided per kg of diet: 4,134 IU vitamin A; 1,653 IU vitamin D; 44 IU vitamin E; 3 mg vitamin K; 0.03 mg vitamin B12; 50 mg niacin; 28 mg pantothenic acid; 8 mg riboflavin.

5 Empirical phytase (ADM, Chicago, IL) included at 1,250 FTU/kg with an estimated release of 0.13% STTD P.

6 Provided per kg of diet: 20 mg copper; 0.30 mg iodine; 110 mg iron; 30 mg manganese; 0.30 mg selenium; 11 mg zinc.

7 Tribasic copper chloride, 58% Cu; SAM Nutrition, Bloomington, MN.

**Table 3:** Analyzed composition of Exp. 1 diets (as-fed basis).^1^

Feed-grade AA:	Low	Moderate	High	High	High
Added N:	None	None	None	DAP^2^	Glycine
SID Lys:CP:	6.0:1	6.5:1	7.0:1	6.5:1	6.5:1
**Nutrient, %**					
** CP**	19.24	19.04	17.21	19.16	18.53
** Moisture**	12.73	13.20	13.04	13.02	12.89
** Crude fat**	2.28	1.97	2.22	2.15	2.38
** Crude fiber**	2.03	1.90	2.02	2.05	2.03
** Ash**	5.55	5.39	5.13	5.06	4.88
** Essential AA**					
** Arg**	1.20	1.17	1.02	0.95	0.97
** His**	0.49	0.50	0.49	0.45	0.48
** Ile**	0.82	0.86	0.82	0.78	0.77
** Leu**	1.59	1.57	1.45	1.39	1.42
** Lys**	1.37	1.46	1.39	1.33	1.33
** Met**	0.47	0.56	0.55	0.49	0.53
** Phe**	0.92	0.91	0.82	0.77	0.78
** Thr**	0.89	0.93	0.93	0.84	0.87
** Trp**	0.27	0.27	0.27	0.28	0.27
** Val**	1.02	1.07	1.02	0.98	0.99
** Non-essential AA**					
** Ala**	0.93	0.92	0.85	0.82	0.85
** Asp**	1.92	1.88	1.66	1.56	1.56
** Cys**	0.29	0.30	0.27	0.24	0.26
** Glu**	3.39	3.34	3.02	2.83	2.86
** Gly**	0.77	0.75	0.68	0.63	1.64
** Pro**	1.09	1.08	1.01	0.96	1.00
** Ser**	0.81	0.78	0.70	0.67	0.70
** Tyr**	0.64	0.63	0.55	0.54	0.55

1 Samples were analyzed for proximate analysis and complete AA profile (University of Missouri Agricultural Experiment Station Chemical Laboratory, Columbia MO).

2 Diammonium phosphate.

Pens of pigs were weighed and feed disappearance was measured at the beginning and end of the study (d 21) to determine average daily gain (ADG), average daily feed intake (ADFI), and gain-to-feed ratio (G:F). Daily feed additions were recorded with a computerized feeding system (FeedPro; Feedlogic Corp., Willmar, MN). Blood samples were collected from pigs on d 21 from four pigs per pen (two barrows and two gilts of medium size) to measure serum blood urea nitrogen (BUN; DetectX Urea Nitrogen Detection Kit; Arbor Assays, Ann Arbor, MI). Pigs were allowed *ad libitum* access to feed before sample collection.

#### Digestibility analysis

Fecal samples were collected on d 21 from three pigs per pen to determine percentage DM. Samples were dried at 55 °C in a forced-air oven for 48 h, and the ratio of dried-to-wet fecal weight determined the fecal DM. Fecal samples were analyzed separately for each pig and the average of the three samples from each pen was then used for statistical analysis. Following fecal DM determination, both ground feed and pen-level pooled fecal samples were dried in a 135 °C oven for 2 h to determine percentage DM of the samples used for titanium analysis (Method 985.01; AOAC International 2007). Fecal samples were analyzed for DM, N, and TiO_2_ at the Kansas State University Swine Laboratory. Titanium dioxide concentration in both dried feed and fecal samples were determined using procedures outlined by Leone (1973). The ATTD of DM and N was calculated using the index method described by Adeola (2001) using the following equation:


$$\text{ATTD}\;\text{DM}\;\text{or}\;N,\;\%=[1-[\left(\frac{{\text{DM}\;\text{or}\;N,\%}_{\text{Fecals}}}{{\text{DM}\;\text{or}\;N,\%}_{\text{Feed}\;}})\times (\frac{{{\text{TiO}}_{2},\%}_{\text{Feed}}}{{{\text{TiO}}_{2},\%}_{\text{Fecals}\;}}\right)]]\times 100$$

### Experiment 2

The objective of Exp. 2 was to evaluate SID Lys:CP ratios in diets formulated to different levels of SID Lys, and thus different dietary SBM inclusion levels. A total of 4,167 pigs (PIC 337 × 1050; initially 13.0 ± 0.27 kg and approximately 21 days after weaning) originating from three sow farms were placed in the research facility over a 6-d period and used in a 14-d study. Pigs were housed in same-sex pens and pens were allotted to 1 of 10 dietary treatments in a randomized complete block design with blocking structure, including sow farm origin, date of entry into the nursery facility, and average pen BW. Two adjacent pens shared a single, double-sided fence line feeder. Each feeder (considered the experimental unit) provided feed for one pen of 26 gilts and one pen of 26 barrows. Composite samples of corn and SBM were analyzed prior to diet formulation (University of Missouri Agricultural Experiment Station Chemical Laboratory) for proximate analysis and complete AA profile (Table 4). Diets were corn-SBM-based and arranged in a 2 × 5 factorial with main effects of SID Lys (1.15 or 1.30% SID Lys) and SID Lys:CP ratio (6.00, 6.22, 6.46, 6.72, and 7.00%) with eight replications per dietary treatment (Table 5). Dietary additions of feed-grade AA were adjusted to meet or exceed NRC (2012) AA requirement estimates in relation to Lys for Met and Cys, Thr, Trp, Val, Ile, and His. Both low and high SID Lys:CP ratio diets, 6.00 and 7.00%, respectively, with low and high SID Lys, were manufactured at the Hord Elevator (Bucyrus, OH) in meal form and blended at the farm by an electronic feeding system (Dry Extract; Big Dutchman, Inc., Holland, MI) to make the intermediate dietary treatments. All diets contained added Zn (830 mg/kg) and Cu (195 mg/kg) for growth promotion. Diet samples were collected and analyzed for proximate analysis and complete AA profile (University of Missouri Agricultural Experiment Station Chemical Laboratory; Table 6).

**Table 4:** Analyzed composition of soybean meal (SBM) and corn for Exp. 2 (as-fed basis).^1^

Nutrient, %	SBM	Corn
** CP**	45.10	7.02
** Dry matter**	88.47	86.52
** Crude fat**	1.42	2.42
** Crude fiber**	3.44	1.68
** Ash**	6.04	1.07
** Essential AA**		
** Arg**	3.32	0.30
** His**	1.23	0.20
** Ile**	2.21	0.25
** Leu**	3.62	0.85
** Lys**	3.02	0.23
** Met**	0.63	0.15
** Phe**	2.45	0.35
** Thr**	1.82	0.25
** Trp**	0.64	0.05
** Val**	2.27	0.33
** Non-essential AA**		
** Ala**	2.01	0.51
** Asp**	5.26	0.46
** Cys**	0.69	0.17
** Glu**	8.60	1.32
** Gly**	1.94	0.26
** Pro**	2.38	0.64
** Ser**	2.03	0.32
** Tyr**	1.73	0.20

1 Samples were analyzed for proximate analysis and complete AA profile (University of Missouri Agricultural Experiment Station Chemical Laboratory, Columbia, MO). These values, using NRC (2012) standardized ileal digestibility coefficients, were then used in diet formulation.

**Table 5:** Diet composition, Exp. 2 (as-fed basis).^1^

SID Lys, %:	1.15		1.30	
SID Lys:CP:	6.00	7.00	6.00	7.00
**Ingredient, %**				
** Corn**	64.73	72.40	58.32	66.86
** Soybean meal**	31.44	22.81	37.94	28.32
** Calcium carbonate**	0.93	0.90	0.94	0.91
** Monocalcium P (21.5% P)**	0.95	1.05	0.85	0.97
** Salt**	0.30	0.30	0.30	0.30
** Liquid Lys (55% Lys)**	0.35	0.75	0.33	0.77
** DL-Met**	0.14	0.22	0.17	0.26
** L-Trp**	0.01	0.06	0.01	0.06
** L-Val**	0.01	0.16	—	0.18
** L-Ile**	—	0.05	—	0.04
** L-Thr**	0.18	0.32	0.19	0.35
** L-His**	—	0.03	—	0.03
** Sodium metabisulfate**	0.50	0.50	0.50	0.50
** Zinc oxide**	0.10	0.10	0.10	0.10
** Copper sulfate**	0.07	0.07	0.07	0.07
** Trace mineral premix^2^**	0.10	0.10	0.10	0.10
** Vitamin premix^3^**	0.10	0.10	0.10	0.10
** Phytase^4^**	0.10	0.10	0.10	0.10
**Total**	100	100	100	100
**Calculated analysis**				
** SID AA**				
** Lys, %**	1.15	1.15	1.30	1.30
** Ile:Lys**	65	56	67	56
** Leu:Lys**	129	110	126	107
** Met:Lys**	35	38	35	38
** Met and Cys:Lys**	58	58	58	58
** Thr:Lys**	65	65	65	65
** Trp:Lys**	19.0	19.2	19.2	19.3
** Val:Lys**	70	70	70	70
** His:Lys**	39.6	34.1	39.8	34.2
** Arg:Lys**	100	78	103	81
** Phe and Tyr:Lys**	126	102	128	104
** Total Lys, %**	1.29	1.27	1.46	1.43
** SID EAA:NEAA**	0.88	0.98	0.88	0.98
** NE, kcal/kg**	2,505	2,530	2,491	2,518
** SID Lys:NE, g/Mcal**	4.59	4.54	5.22	5.16
** CP, %**	19.2	16.4	21.7	18.6
** Ca, %**	0.66	0.64	0.66	0.65
** STTD P, %**	0.47	0.47	0.47	0.47
** Ca:P**	1.10	1.10	1.10	1.10

1 Diets were fed from 13.0 to 26.4 kg.

2 Provided per kg of diet: 20 mg copper; 0.40 mg iodine; 110 mg iron; 30 mg manganese; 0.30 mg selenium; 110 mg zinc.

3 Provided per kg of diet: 3,307 IU vitamin A; 1,323 IU vitamin D; 35 IU vitamin E; 3 mg vitamin K; 0.03 mg vitamin B12; 44 mg niacin; 22 mg pantothenic acid; 6 mg riboflavin; 6 mg thiamin.

4 Quantum Blue 2G (AB Vista, Marlborough, Wiltshire, UK) included at 2,000 FTU/kg with an estimated release of 0.14% STTD P.

**Table 6:** Analyzed composition of Exp. 2 diets (as-fed basis).^1^

SID Lys, %:	1.15					1.30				
SID Lys:CP:	6.00	6.22	6.46	6.72	7.00	6.00	6.22	6.46	6.72	7.00
**Nutrient, %**										
** CP**	19.41	19.24	17.60	17.31	16.37	22.37	20.45	20.11	19.09	18.97
** Dry matter**	87.68	87.95	87.15	87.15	87.10	87.71	87.63	87.55	87.58	87.37
** Crude fat**	1.24	1.39	1.55	1.54	1.64	1.16	1.13	1.03	1.36	1.38
** Crude fiber**	2.18	2.07	2.06	1.87	1.82	2.04	1.98	1.90	1.93	2.02
** Ash**	4.78	4.81	4.68	4.76	4.53	5.15	4.97	5.14	4.64	4.60
** Essential AA**										
** Arg**	1.22	1.18	1.07	1.03	1.01	1.47	1.28	1.27	1.18	1.16
** His**	0.50	0.50	0.46	0.45	0.45	0.59	0.53	0.53	0.51	0.52
** Ile**	0.85	0.83	0.77	0.77	0.75	1.01	0.90	0.90	0.86	0.85
** Leu**	1.60	1.57	1.47	1.43	1.41	1.86	1.67	1.66	1.58	1.57
** Lys**	1.27	1.29	1.24	1.28	1.27	1.48	1.38	1.43	1.38	1.41
** Met**	0.42	0.43	0.42	0.48	0.50	0.49	0.49	0.54	0.51	0.55
** Phe**	0.94	0.91	0.84	0.82	0.80	1.12	0.99	0.98	0.92	0.90
** Thr**	0.85	0.85	0.84	0.85	0.86	0.98	0.94	0.98	0.91	0.93
** Trp**	0.19	0.21	0.19	0.20	0.20	0.24	0.21	0.23	0.23	0.21
** Val**	0.96	0.97	0.94	0.94	0.94	1.11	1.03	1.06	1.06	1.09
** Non-essential AA**										
** Ala**	0.93	0.92	0.86	0.83	0.83	1.07	0.97	0.95	0.92	0.91
** Asp**	1.90	1.84	1.68	1.62	1.57	2.31	2.00	1.99	1.84	1.82
** Cys**	0.31	0.30	0.28	0.27	0.27	0.35	0.31	0.31	0.30	0.30
** Glu**	3.45	3.37	3.12	3.02	2.94	4.11	3.64	3.60	3.39	3.34
** Gly**	0.77	0.75	0.70	0.67	0.66	0.91	0.81	0.80	0.75	0.74
** Pro**	1.08	1.05	1.00	0.98	0.97	1.21	1.11	1.10	1.07	1.06
** Ser**	0.77	0.75	0.70	0.67	0.64	0.89	0.80	0.79	0.75	0.72
** Tyr**	0.64	0.61	0.58	0.56	0.55	0.74	0.67	0.66	0.62	0.62

1 Samples were analyzed for proximate analysis and complete AA profile (University of Missouri Agricultural Experiment Station Chemical Laboratory, Columbia, MO).

Pens of pigs were weighed and feed disappearance was measured at the beginning and end of the study (d 14) to determine ADG, ADFI, and G:F. Daily feed additions were recorded by the electronic feeding system.

### Experiment 3

Experiment 3 was designed to evaluate SID Lys:CP ratios in diets containing different basal levels of SBM with or without DDGS as a different and alternative source of CP. This was completed by using 15% DDGS to create high and low levels of SBM within the differing SID Lys:CP ratios. The objective was to evaluate a different source of CP from a readily available source, DDGS, in combination with SBM. A total of 5,059 (PIC 800 × Camborough and DNA 600 × 241; initially 11.0 ± 0.90 kg and approximately 19 days after weaning) originating from two sow farms were placed in two rooms at the research facility and used in an 18-d study. Pigs were housed in mixed-sex pens with approximately 35 pigs per pen, and pens of pigs were allotted to 1 of 12 dietary treatments in a completely randomized design when pigs reached approximately 11 kg. Composite samples of corn, SBM, and DDGS were collected prior to diet formulation and analyzed for proximate analysis and complete AA profile (University of Missouri Agricultural Experiment Station Chemical Laboratory; Table 7). Treatment diets were corn-SBM-based and arranged in a 2 × 6 factorial with main effects of DDGS (0 or 15%) and SID Lys:CP ratio (6.01, 6.22, 6.45, 6.70, 6.97, or 7.26%; Table 8). There were 12 replications per treatment except for treatment 5 (SID Lys:CP of 6.97% with no DDGS), which had 11 replications. Dietary additions of feed-grade AA were adjusted to meet or exceed NRC (2012) AA requirement estimates in relation to Lys for Met and Cys, Thr, Trp, Val, Ile, and His. Both the low and high SID Lys:CP ratio diets, 6.01 and 7.26%, respectively, with and without DDGS, were manufactured (Hubbard Feeds, Mankato, MN) in meal form and blended at the farm by an electronic feeding system (Dry Extract; Big Dutchman, Inc., Holland, MI) to make intermediate dietary treatments. Diet samples were collected and analyzed for proximate analysis and complete AA profile (University of Missouri Agricultural Experiment Station Chemical Laboratory; Table 9).

**Table 7:** Analyzed composition of corn, soybean meal (SBM), and dried distillers grains with solubles (DDGS) for Exp. 3 (as-fed basis).^1^

Nutrient, %	Corn	SBM	DDGS
** CP**	6.76	45.64	28.50
** Dry matter**	85.66	89.76	88.89
** Crude fat**	2.62	1.98	8.30
** Crude fiber**	1.61	4.08	7.53
** Ash**	1.10	6.51	4.96
** Essential AA**			
** Arg**	0.34	3.30	1.28
** His**	0.20	1.22	0.81
** Ile**	0.25	2.27	1.07
** Leu**	0.77	3.59	3.05
** Lys**	0.27	3.02	0.96
** Met**	0.15	0.67	0.49
** Phe**	0.33	2.43	1.31
** Thr**	0.25	1.80	1.07
** Trp**	0.05	0.63	0.21
** Val**	0.34	2.36	1.42
** Non-essential AA**			
** Ala**	0.50	2.04	1.80
** Asp**	0.49	5.33	1.77
** Cys**	0.16	0.71	0.54
** Glu**	1.25	8.65	3.73
** Gly**	0.29	1.99	1.09
** Pro**	0.62	2.39	2.04
** Ser**	0.30	1.98	1.18
** Tyr**	0.23	1.76	1.03

1 Samples were analyzed for proximate analysis and complete AA profile (University of Missouri Agricultural Experiment Station Chemical Laboratory, Columbia, MO). These values, using NRC (2012) standardized ileal digestibility coefficients, were then used in diet formulation.

**Table 8:** Diet composition, Exp. 3 (as-fed basis).^1^

	No DDGS		15% DDGS	
SID Lys:CP:	6.01	7.26	6.01	7.26
**Ingredient, %**				
** Corn**	59.03	69.18	52.99	63.18
** Soybean meal**	37.79	26.48	28.70	17.24
** Corn DDGS (8.3% oil)**	—	—	15.00	15.00
** Calcium carbonate**	0.95	0.92	1.04	1.00
** Monocalcium P (21.5% P)**	0.85	1.05	0.65	0.83
** Salt**	0.60	0.61	0.54	0.55
** L-Lys**	0.21	0.57	0.43	0.79
** DL-Met**	0.15	0.27	0.16	0.27
** L-Thr**	0.15	0.31	0.19	0.35
** L-Trp**	0.01	0.07	0.04	0.10
** L-Val**	—	0.18	0.01	0.23
** L-Ile**	—	0.06	—	0.14
** L-His**	—	0.05	—	0.08
** Trace mineral premix^2^**	0.08	0.08	0.08	0.08
** Vitamin premix^3^**	0.05	0.05	0.05	0.05
** Copper sulfate**	0.07	0.07	0.07	0.07
** Selenium^4^**	0.05	0.05	0.05	0.05
** Phytase^5^**	0.02	0.02	0.02	0.02
**Total**	100	100	100	100
**Calculated analysis**				
** SID AA, %**				
** Lys, %**	1.30	1.30	1.30	1.30
** Ile:Lys**	68	56	62	56
** Leu:Lys**	122	100	127	104
** Met:Lys**	35	39	35	39
** Met and Cys:Lys**	58	58	58	58
** Thr:Lys**	65	65	65	65
** Trp:Lys**	19	19	19	19
** Val:Lys**	72	70	70	70
** His:Lys**	39	34	38	34
** Arg:Lys**	104	79	93	67
** Phe and Tyr:Lys**	128	100	122	93
** Total Lys, %**	1.47	1.44	1.49	1.46
** SID EAA:NEAA**	0.88	0.99	0.92	1.07
** NE, kcal/kg**	2,504	2,537	2,489	2,526
** SID Lys:NE, g/Mcal**	5.19	5.12	5.22	5.15
** CP, %**	21.6	17.9	21.6	17.9
** Ca, %**	0.67	0.65	0.65	0.63
** STTD P, %**	0.45	0.46	0.45	0.45
** Ca:P**	1.10	1.10	1.10	1.10

1 Diets were fed from 11.0 to 20.4 kg.

2 Provided per kg of diet: 10 mg copper; 0.53 mg iodine; 110 mg iron; 30 mg manganese; 130 mg zinc.

3 Provided per kg of diet: 12,100 IU vitamin A; 2,200 IU vitamin D3; 66 IU vitamin E; 7 mg vitamin K; 0.06 mg vitamin B12; 50 mg niacin; 45 mg pantothenic acid; 9 mg riboflavin; 1 mg thiamin.

4 Provided per kg of diet: 0.30 mg Se.

5 Quantum Blue 5G (AB Vista, Marlborough, Wiltshire, UK) included at 751 FTU/kg with an estimated release of 0.12% STTD P.

**Table 9:** Analyzed composition of Exp. 3 diets (as-fed basis).^1^

	No DDGS						DDGS					
SID Lys:CP:	6.01	6.22	6.45	6.70	6.97	7.26	6.01	6.22	6.45	6.70	6.97	7.26
**Nutrient, %**												
** CP**	22.70	20.94	19.95	19.69	19.44	18.55	21.81	21.08	19.94	19.86	18.73	18.73
** Dry matter**	87.73	87.84	87.83	87.66	87.57	87.88	88.63	88.48	88.63	88.45	88.25	88.36
** Crude fat**	1.90	2.04	2.17	1.75	1.53	1.69	2.52	2.70	2.88	2.69	3.02	3.01
** Crude fiber**	2.36	2.40	2.40	1.89	1.70	1.72	2.77	2.74	2.69	3.07	2.80	2.93
** Ash**	5.33	4.93	5.02	5.81	5.03	4.50	5.68	5.35	5.30	5.43	5.22	5.32
** Essential AA**												
** Arg**	1.48	1.36	1.30	1.23	1.21	1.12	1.31	1.26	1.21	1.15	1.05	1.05
** His**	0.63	0.59	0.59	0.57	0.56	0.53	0.61	0.60	0.60	0.59	0.57	0.60
** Ile**	1.01	0.94	0.91	0.90	0.87	0.81	0.95	0.91	0.92	0.88	0.85	0.86
** Leu**	1.84	1.75	1.68	1.63	1.59	1.53	1.96	1.90	1.86	1.78	1.71	1.73
** Lys**	1.50	1.44	1.44	1.51	1.52	1.42	1.53	1.49	1.51	1.46	1.46	1.51
** Met**	0.41	0.45	0.39	0.44	0.46	0.42	0.49	0.47	0.46	0.44	0.43	0.46
** Phe**	1.14	1.06	1.01	0.98	0.96	0.89	1.10	1.06	1.02	0.97	0.91	0.91
** Thr**	1.00	0.92	0.94	0.96	0.97	0.93	0.99	0.99	0.97	0.94	0.90	0.95
** Trp**	0.26	0.25	0.27	0.27	0.25	0.25	0.25	0.26	0.27	0.26	0.27	0.26
** Val**	1.10	1.08	1.04	1.05	1.06	1.01	1.07	1.08	1.09	1.05	1.05	1.06
** Non-essential AA**												
** Ala**	1.07	1.02	0.98	0.94	0.92	0.89	1.15	1.13	1.10	1.05	1.01	1.03
** Asp**	2.32	2.12	2.03	1.94	1.91	1.74	2.05	1.96	1.87	1.75	1.63	1.62
** Cys**	0.37	0.35	0.33	0.32	0.31	0.30	0.38	0.38	0.36	0.35	0.33	0.35
** Glu**	4.10	3.82	3.66	3.53	3.46	3.24	3.88	3.75	3.66	3.43	3.25	3.26
** Gly**	0.93	0.86	0.82	0.78	0.77	0.73	0.88	0.86	0.83	0.78	0.73	0.74
** Pro**	1.24	1.19	1.14	1.10	1.08	1.05	1.35	1.32	1.28	1.23	1.19	1.21
** Ser**	0.97	0.90	0.87	0.82	0.80	0.77	0.93	0.92	0.86	0.80	0.78	0.79
** Tyr**	0.79	0.74	0.72	0.69	0.67	0.63	0.78	0.76	0.73	0.70	0.66	0.67

1 Samples were analyzed for proximate analysis and complete AA profile (University of Missouri Agricultural Experiment Station Chemical Laboratory, Columbia, MO).

Pens of pigs were weighed and feed disappearance was measured at the beginning and end of the study (d 18) to calculate ADG, ADFI, and G:F. Daily feed additions were recorded by the electronic feeding system.

### Statistical analysis

#### Experiment 1

Growth, digestibility, and fecal DM data were analyzed as a randomized complete block design for one-way ANOVA using the lmer function from the lme4 package in R Studio (Version 4.3.1, R Core Team, Vienna, Austria) with pen serving as the experimental unit, dietary treatment as a fixed effect, and BW block as a random intercept. Linear and quadratic contrasts were tested within increasing levels of feed-grade AA without addition of added DAP or Gly. The effect of DAP and Gly were tested using pairwise comparisons to compare their responses to moderate and high levels of feed-grade AA individually. Blood urea N data were analyzed with pig serving as the observational unit, dietary treatment and gender as fixed effects, and block and microtiter plates as random intercepts. When treatment was a significant source of variation, differences were determined by pairwise comparison using the Tukey-Kramer multiplicity adjustment to control for Type I Error. For all experiments, results were considered significant with *P *≤ 0.05 and were considered marginally significant with 0.05 < *P *≤ 0.10.

#### Experiments 2 and 3

Data for Exp. 2 and 3 were analyzed as a randomized complete block design and a completely randomized design, respectively, for one-way ANOVA using the lmer function from the lme4 package in R Studio (Version 4.3.1, R Core Team, Vienna, Austria). For Exp. 2, feeder (two pens of pigs) was considered the experimental unit. Treatment was included in the model as a fixed effect, and block was included in the model as a random intercept, which incorporated initial pen BW, sow farm origin, and date of entry into the nursery facility. For Exp. 3, pen was considered the experimental unit. Treatment was included in the model as a fixed effect, and nursery entry date and room were included in the model as random effects to account for differences in genetic lines and fill date. Contrast statements were used to evaluate the interactive effect of SID Lys:CP ratio × SID Lys and SID Lys:CP ratio × DDGS for Exp. 2 and 3, respectively, as well as their main effects.

Dose–response curves were created using quadratic polynomial (QP) and broken-line linear (BLL) models in SAS OnDemand for Academics (SAS Institute, Inc., Cary, NC) using the NLMIXED and GLIMMIX procedures, respectively. Following model fit, the best-fitting model was selected using the Bayesian Information Criterion (BIC). A decrease in BIC greater than 2.0 among models for a particular response criterion was considered an improved fit. For Exp. 2, a broken line linear analysis was conducted for the effect of SID Lys:CP ratio on G:F in the low SID Lys diets (1.15%), which is below the SID Lys requirement estimate. For Exp. 3, QP analyses were conducted for the effect of SID Lys:CP ratio on G:F within diets with and without 15% DDGS.

## Results

### Experiment 1

Overall (d 0 to 21), there was no evidence of differences in ADG due to dietary treatment (Table 10). A decrease in G:F was observed (linear, *P *= 0.002; quadratic, *P *= 0.054), with the greatest change occurring as the SID Lys:CP ratio increased from 6.5 to 7.0%. Incorporating either DAP or Gly to the high feed-grade AA diet led to an increase (*P *= 0.003 and *P *= 0.001, respectively) in G:F compared to the pigs fed the high feed-grade AA diet due to a reduction (*P *= 0.007) or numerical reduction (*P *= 0.109), respectively, in ADFI. No differences were observed between either DAP or Gly and the moderate feed-grade AA diet.

**Table 10:** Effect of feed-grade amino acid (AA) inclusion with and without an added nitrogen (N) source on growth performance, serum blood urea nitrogen (BUN), fecal dry matter (DM), and digestibility of DM and crude protein (CP; Exp. 1).^1^

Feed-grade AA:	Low	Moderate	High	High	High		*P* =			
Added N:	None	None	None	DAP^2^	Glycine		SID Lys:CP^3^			
SID Lys:CP:	6.0:1	6.5:1	7.0:1	6.5:1	6.5:1	SEM	Linear	Quadratic	DAP^4^	Glycine^5^
**BW, kg**										
** d 0**	10.3	10.3	10.3	10.4	10.4	0.19	0.715	0.649	0.354	0.297
** d 21**	22.2	22.1	22.0	21.8	22.1	0.32	0.344	0.965	0.377	0.518
**Overall (d 0 to 21)**										
** ADG, g**	562	560	557	545	560	8.8	0.616	0.989	0.280	0.736
** ADFI, g**	841	838	864	818	838	14.3	0.163	0.291	0.007	0.109
** G:F, g/kg**	668	669	645	667	669	4.9	0.002	0.054	0.003	0.001
**Blood urea N, mg/dL**	8.17	7.12	6.27	6.88	6.76	0.224	< 0.001	0.680	0.038	0.091
**Fecal DM, %**	20.49	19.61	21.99	24.81	22.87	0.660	0.118	0.051	0.005	0.351
**ATTD DM, %**	83.88	84.08	83.82	85.55	84.70	0.630	0.951	0.766	0.060	0.328
**ATTD CP, %^6^**	78.69	77.00	75.43	81.78	79.12	1.127	0.048	0.963	< 0.001	0.026

1 A total of 981 pigs (initially 10.3 ± 0.19 kg) were used in a 21-d growth study with 19 to 20 pigs per pen and 10 replicates per treatment.

2 DAP = diammonium phosphate.

3 Comparing the main effects of low, moderate, and high feed-grade AA inclusion without added N.

4 Pairwise comparison between high feed-grade AA + DAP and high feed-grade AA.

5 Pairwise comparison between high feed-grade AA + glycine and high feed-grade AA.

6 DAP to moderate feed-grade AA (*P *= 0.005).

There was a decrease (linear, *P *< 0.001) in BUN as the SID Lys:CP ratio increased. Adding nitrogen to the high feed-grade AA diet in the form of DAP or Gly increased (*P = *0.038) or tended to increase (*P = *0.091) BUN, respectively, with no differences when compared to the moderate feed-grade AA diet.

There was a tendency (*P *= 0.051) for a quadratic effect in fecal DM with pigs fed the moderate feed-grade AA diet having the lowest fecal DM. Pigs fed the diet containing DAP had increased (*P *= 0.005) fecal DM compared with the pigs fed the high feed-grade AA diet; however, there was no difference in pigs fed Gly.

There was a tendency for improvement (*P *= 0.060) in ATTD of DM for pigs fed the diet containing DAP compared to pigs fed the high feed-grade AA diet. Additionally, ATTD of CP decreased (linear, *P *= 0.048) with increasing feed-grade AA. Pigs fed diets containing either DAP or Gly had increased (*P *< 0.001 and *P *= 0.026, respectively) CP digestibility compared to pigs fed the high feed-grade AA diet. Pigs fed the diet containing DAP had increased (*P *= 0.005) CP digestibility compared to the pigs fed the moderate feed-grade AA diet.

### Experiment 2

**Figure 1:**
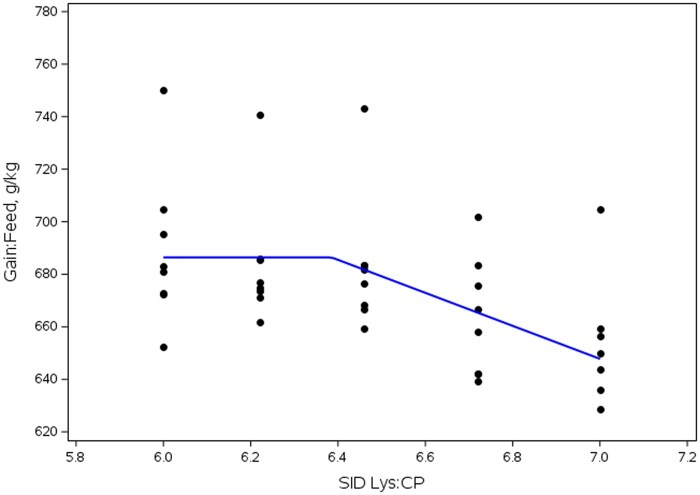
Influence of SID Lys:CP ratio on feed efficiency in Exp. 2. Breakpoint analysis indicates inflection point was observed at SID Lys:CP of 6.39% in 1.15% SID Lys diets.

Overall (d 0 to 14), a SID Lys:CP × SID Lys interaction was observed (linear, *P *< 0.001; Table 11) for G:F where increasing the SID Lys:CP ratio decreased G:F in both 1.15 and 1.30% SID Lys levels, with a greater magnitude of reduction observed in pigs fed diets formulated to 1.15% SID Lys (quadratic, *P *= 0.016) than in diets formulated to 1.30% SID Lys (linear, *P *< 0.001). An increase in ADG and d 14 BW was observed (*P *≤ 0.002) in pigs fed 1.30% SID Lys compared to those fed 1.15% SID Lys. Additionally, as SID Lys:CP ratio increased, ADFI increased (*P *= 0.004; Table 12). Dose response analysis indicated that maximum G:F was observed at 6.39% SID Lys:CP for the diets formulated to 1.15% SID Lys (Figure 1).

**Table 11:** Interactive effects of standardized ileal digestible (SID) Lys to crude protein (CP) ratio and SID Lys on growth performance (Exp. 2).^1^

	SID Lys, %											*P* =		
	1.15					1.30						SID Lys:CP × SID Lys		
SID Lys:CP:	6.00	6.22	6.46	6.72	7.00	6.00	6.22	6.46	6.72	7.00	SEM	Linear	Quadratic	SID Lys^2^
**BW, kg**														
** d 0**	13.0	13.0	13.0	12.9	13.0	12.9	13.2	13.0	13.1	13.0	0.27	0.474	0.207	0.230
** d 14**	21.3	21.2	21.2	21.1	21.1	21.3	21.9	21.6	21.6	21.5	0.40	0.602	0.253	0.002
**Overall (d 0 to 14)**														
** ADG, g**	588	582	588	586	579	600	617	609	601	604	12.3	0.917	0.720	< 0.001
** ADFI, g**	855	851	862	884	894	846	866	862	867	866	21.4	0.165	0.319	0.291
** G:F, g/kg^3^**	689	684	683	664	649	709	713	708	695	698	9.8	< 0.001	0.133	< 0.001

1 A total of 4,167 pigs (initially 13.0 ± 0.27 kg) were used in a 14-d growth study with 26 pigs per pen (52 pigs per feeder) and eight replications per treatment.

2 Main effect of SID Lys.

3 Quadratic effect of SID Lys:CP in 1.15% SID Lys, *P *= 0.016. Quadratic effect of SID Lys:CP in 1.30% SID Lys, *P *= 0.777; Linear effect of SID Lys:CP in 1.15% SID Lys, *P *< 0.001. Linear effect of SID Lys:CP in 1.30% SID Lys, *P *< 0.001.

**Table 12:** Main effects of standardized ileal digestible (SID) Lys to crude protein (CP) ratio on growth performance (Exp. 2).^1^

	SID Lys:CP						*P* =	
	6.00	6.22	6.46	6.72	7.00	SEM	Linear	Quadratic
**BW, kg**								
** d 0**	13.0	13.1	13.0	13.0	13.0	0.25	0.944	0.704
** d 14**	21.3	21.5	21.4	21.3	21.3	0.38	0.731	0.418
**Overall (d 0 to 14)**								
** ADG, g**	594	599	598	594	591	10.9	0.502	0.397
** ADFI, g**	851	859	862	875	880	19.7	0.004	0.953
** G:F, g/kg**	699	698	695	679	673	9.5	< 0.001	0.056

1 A total of 4,167 pigs (initially 13.0 ± 0.27 kg) were used in a 14-d growth study with approximately 26 pigs per pen (52 pigs per feeder).

### Experiment 3

**Figure 2:**
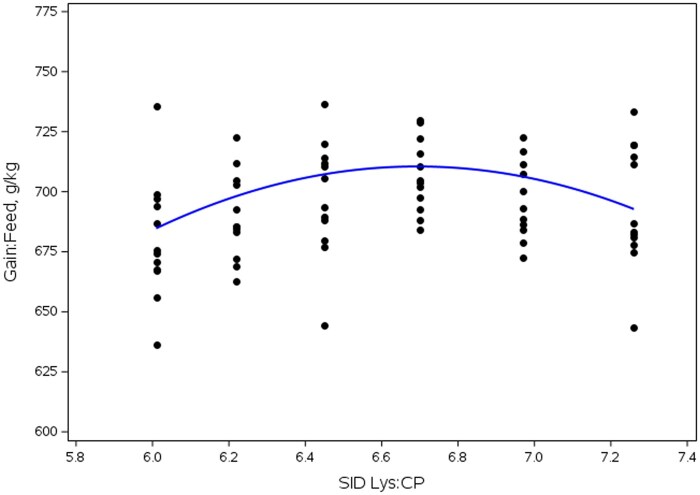
Influence of SID Lys:CP ratio on feed efficiency in diets without DDGS in Exp. 3. Maximum G:F was observed at SID Lys:CP of 6.69%.

**Figure 3:**
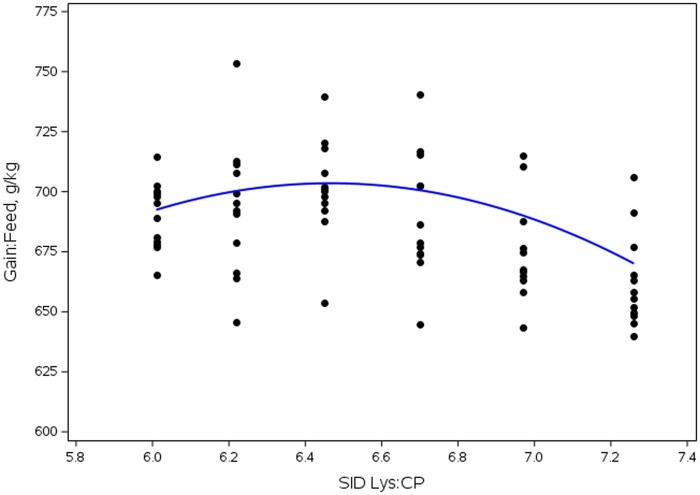
Influence of SID Lys:CP ratio on feed efficiency in diets with 15% DDGS in Exp. 3. Maximum G:F was observed at SID Lys:CP of 6.47%.

Overall (d 0 to 18), a SID Lys:CP ratio × DDGS interaction was observed (linear, *P *< 0.001) for G:F (Table 13) driven by differences in SID Lys:CP in 0 and 15% DDGS. Pigs fed diets without DDGS had decreased G:F when fed diets with the lowest and highest SID Lys:CP ratios (quadratic, *P *< 0.001); whereas in pigs fed diets with DDGS, G:F decreased (quadratic, *P *= 0.002) as the SID Lys:CP ratio increased above 6.45%. For main effects (Table 14), ADG increased then decreased (quadratic, *P *= 0.021) as the SID Lys:CP ratio increased, with the greatest improvement in ADG observed in pigs fed diets with ratios of 6.45 to 6.97%. Additionally, ADFI increased (linear, *P *= 0.018) as SID Lys:CP increased. Pigs fed diets without DDGS tended to have increased (*P *≤ 0.071) ADG. Dose response analysis indicated that maximum G:F was observed at 6.69% SID Lys:CP for the corn-soy diets (0% DDGS; Figure 2); whereas in 15% DDGS diets (Figure 3), maximum G:F was observed at 6.47% SID Lys:CP.

**Table 13:** Interactive effects of standardized ileal digestible (SID) Lys to crude protein (CP) ratio and dried distillers grains with solubles (DDGS) on growth performance (Exp. 3).^1^

	No DDGS						15% DDGS							SID Lys:CP × DDGS, *P* =	
SID Lys:CP:	6.01	6.22	6.45	6.70	6.97	7.26	6.01	6.22	6.45	6.70	6.97	7.26	SEM	Linear	Quadratic
**BW, kg**															
** d 0**	10.9	11.0	11.0	11.0	10.9	11.2	11.0	11.1	11.0	10.9	11.1	10.9	0.90	0.511	0.745
** d 18**	20.1	20.4	20.4	20.6	20.6	20.7	20.1	20.2	20.7	20.4	20.4	19.9	1.28	0.267	0.464
**Overall (d 0 to 18)**															
** ADG, g** ^2^	506	521	514	529	534	524	499	503	530	517	513	500	22.5	0.282	0.358
** ADFI, g**	742	748	732	744	763	760	720	722	752	748	756	751	45.3	0.540	0.219
** G:F, g/kg** ^3^ ^,^ ^4^	683	699	704	713	702	692	696	700	708	694	682	669	13.6	< 0.001	0.437

1 A total of 5,059 pigs (initially 11.0 ± 0.90 kg) were used in an 18-d growth study with approximately 35 pigs per pen and 11 or 12 replications per treatment.

2,3 Main effect of DDGS, ^2^*P *= 0.071. ^3^*P *= 0.010.

4 Quadratic effect of SID Lys:CP in no DDGS, *P *< 0.001. Linear (*P *< 0.001) and quadratic (*P *= 0.002) effect of SID Lys:CP in 15% DDGS diets.

**Table 14:** Main effects of standardized ileal digestible (SID) Lys to crude protein (CP) on growth performance (Exp. 3).^1^

	SID Lys:CP							*P* =	
	6.01	6.22	6.45	6.70	6.97	7.26	SEM	Linear	Quadratic
**BW, kg**									
** d 0**	11.0	11.0	11.0	11.0	11.0	11.0	0.89	0.895	0.971
** d 18**	20.1	20.3	20.5	20.5	20.5	20.3	1.26	0.432	0.145
**Overall (d 0 to 18)**									
** ADG, g**	503	512	522	523	524	512	21.3	0.211	0.021
** ADFI, g**	731	735	742	746	760	755	44.0	0.018	0.714
** G:F, g/kg**	690	699	706	703	692	680	13.1	0.007	< 0.001

1 A total of 5,059 pigs (initially 11.0 ± 0.90 kg) were used in an 18-d growth study with approximately 35 pigs per pen.

## Discussion

As the swine industry continues to prioritize more sustainable and efficient production, optimizing N efficiency has become a key focus area. One of the major environmental concerns associated with pig production is N excretion, which is largely influenced by dietary CP. High CP diets significantly contribute to N waste, as excess protein that is not used for growth is excreted primarily in the form of urea and ammonia (Millet et al. 2018). A review of 33 swine metabolism studies suggests that N excretion decreases by approximately 8% for every 1 percentage unit reduction in CP, regardless of pig body weight (Kerr 2003). Therefore, low protein, feed-grade AA fortified diets are widely used in swine production. Lowering CP in swine diets has been shown to improve gut health by decreasing the incidence of diarrhea (Limbach et al. 2021), minimizing N excretion (Portejoie et al. 2004), and reducing feed costs by decreasing the amount of intact protein sources in diet. With the commercial availability of feed-grade AA, it is possible to reduce dietary CP while still meeting EAA requirements. However, adequate N must still be provided to support the synthesis of NEAA. Therefore, formulating diets based on an optimal SID Lys:CP ratio could serve as a method to ensure sufficient N availability for NEAA synthesis, thereby maintaining pig performance.

Rocha et al. (2022) conducted a meta-analysis including data from 23 nursery experiments with pigs ranging from 5 to 36 kg and identified a SID Lys:CP ratio of 6.6% as the threshold above which ADG and G:F begin to decline. Although this meta-analysis combines studies involving pigs of varying body weights, the findings closely align with results from the present studies, in which maximum G:F was observed at a SID Lys:CP level of approximately 6.5%. Previously, Millet et al. (2018) evaluated the effects of CP and two dietary Lys levels that were intentionally formulated below the optimum level to ensure that Lys was the first limiting AA. They reported an optimal SID Lys:CP ratio of 6.4% for pigs ranging from 8.3 kg to approximately 21.7 kg, above which growth performance declined. The findings of Millet et al. (2018) are consistent with the breakpoint analysis from Exp. 2, where optimal G:F was observed at a SID Lys:CP ratio of 6.39%. Overall, if the SID Lys:CP ratio is high and dietary CP is insufficient to supply adequate N, growth performance, particularly feed efficiency, can be compromised (Mansilla 2013). A potential explanation for this reduction in growth performance is that the limited N provided from dietary CP prevents adequate synthesis of NEAA (Peng et al. 2016).

NPN sources are rarely used in swine as monogastric animals cannot efficiently utilize NPN in the same manner as ruminants. However, NPN utilization as a strategy to limit N excretion has been of interest. Mansilla (2013) observed that G:F was reduced in pigs fed a diet deficient in NEAA compared to those supplemented with NPN in the form of urea and ammonium salts, showing that N from ammonia can be used as efficiently as intact protein as a source of N for NEAA synthesis. Urea has been the most studied NPN source in swine; however, ammonium salts have also been researched. Mansilla et al. (2017) demonstrated that a diet containing ammonium salts (diammonium phosphate; DAP at 1.2% and diammonium citrate; DAC at 1.7%) can effectively serve as a N source in a low protein diet. Mansilla et al. (2017) observed that increasing NPN inclusion improved G:F, with no differences observed between a high-NPN diet and the diet in which N was provided from intact protein sources. AA can also be used as a N source for the synthesis of other NEAA by serving as a N donor during transamination reactions (Rezaei et al. 2013). Based on the findings of Exp. 1, growth performance can be maintained when a low protein diet, high in feed-grade AA is supplemented with a N source like DAP or Gly to support the synthesis of NEAA. These results agree with the work conducted by Mansilla et al. (2013; 2017).

Blood urea nitrogen serves as an indicator of protein metabolism and N balance, as urea is the main nitrogenous product of protein catabolism (Pedersen and Boisen 2001). Consequently, BUN concentrations are directly affected by dietary CP level and AA in the diet. Elevated BUN levels typically suggest excessive dietary protein intake or an imbalance in AA composition, resulting in increased N excretion, while lower BUN concentrations can indicate improved N utilization or an inadequate supply of NEAA-N. In Exp. 1, BUN concentrations decreased as the SID Lys:CP ratio increased, indicating that at low SID Lys:CP ratios, there was greater total N (CP) intake and therefore greater AA oxidation. Because blood was collected once in the morning, any postprandial variation in BUN would be consistent among treatments and therefore would not impact the interpretation of relative dietary effects, although absolute BUN concentrations may have been influenced by fed-state conditions. This approach is consistent with previous nursery pig studies that evaluated BUN concentrations under *ad libitum* conditions (Waguespack et al. 2011; Nørgaard et al. 2013).

Millet et al. (2018) similarly observed reductions in serum urea N concentrations with decreasing dietary CP. Additionally, in Exp. 1, pigs fed diets containing DAP and Gly had greater BUN concentrations compared to those fed the high feed-grade AA diet, which also contained the lowest CP. This observation is logical, as DAP and Gly supply N, thus increasing the calculated CP of the diet. Interestingly, Whang and Easter (2000) reported that BUN ­concentrations are negatively correlated with G:F, indicating higher BUN was associated with poorer G:F. This disagrees with the results from Exp. 1 where G:F decreased as the SID Lys:CP ratio increased. At high SID Lys:CP ratios, dietary N may become insufficient for the synthesis of NEAA, resulting in a deficiency that can decrease growth performance. In such cases, pigs utilize the available N as efficiently as possible until NEAA become limiting, which is often reflected by a reduction in BUN concentrations.

Previous literature indicates that reducing CP in swine diets improves fecal consistency. Excess dietary CP can result in undigested protein fermentation in the hindgut, increasing microbial activity (Gao et al. 2020) and potentially leading to incidences of diarrhea (Marchetti et al. 2023). Htoo et al. (2007) observed that a low CP diet supplemented with AA reduced the proliferation of potentially harmful bacteria, which may explain the findings from Exp. 1, where the low CP diet tended to have the highest fecal DM. Similarly, other studies have reported a decrease in diarrhea in pigs fed lower CP diets (Heo et al. 2009; Limbach et al. 2021). Interestingly, when DAP was added to the high feed-grade AA diet, fecal DM was increased. Engelsmann et al. (2023) observed that higher feed intake was associated with a higher probability of diarrhea. This increase in fecal DM observed in pigs fed the diet supplemented with DAP may be attributed to the reduction in ADFI for this treatment relative to the high feed-grade AA diet. Another potential explanation may be the digestibility of DAP relative to intact protein. In the current study, pigs fed the DAP-supplemented diet had greater ATTD of CP than those fed diets without a supplemental N source. The N from DAP may be more bioavailable because it bypasses the need for proteolytic digestion required by intact proteins. As a result, less N reaches the hindgut for microbial fermentation, potentially reducing diarrhea incidence.

It is important to note that N digestibility in the current study, which was then converted to a CP basis, was determined using ATTD rather than SID. ATTD for AA is generally considered less accurate as it does not account for endogenous losses (Stein et al. 2007). However, Mansilla (2013) suggested that fecal digestibility values are more accurate for estimating N bioavailability compared to SID values. This is because N absorbed by the lower gut, primarily in the form of ammonia due to microbial fermentation, can be efficiently absorbed by the mucosa in the lower gut for body protein deposition (Mansilla 2013). Mansilla (2013) observed that the ATTD of DM was greater for pigs with a high urea-N infusion than those infused with saline. Although pigs were not infused in the present study, the ATTD of DM tended to be ­higher in pigs fed DAP compared to those on the high feed-grade AA diet without added N. Mansilla (2013) had noted enhanced N utilization for pigs infused with urea, which likely improved overall nutrient absorption and utilization, including DM. Additionally, in Exp. 1, a linear reduction in ATTD of CP was observed with increasing SID Lys:CP or lowering CP in the diets. Petersen et al. (2014) observed that corn has decreased SID CP (80.1%) compared to SBM (85.9%). Therefore, a potential explanation for this response could be the change in diet composition. As the SID Lys:CP ratio increases, corn inclusion, which is less digestible compared to SBM, increases, while SBM decreases. Furthermore, when DAP and Gly were supplemented to the high feed-grade AA diet without added N, there was an increase in the ATTD of CP, likely because these N sources are more bioavailable than N provided by an intact protein source. Intact proteins must undergo proteolytic digestion by enzymes to be absorbed (Rivest et al. 2000). Because N from NPN or amino N are already in a simplified form, there may be quicker absorption in the gastrointestinal tract.

Based on NRC (2012), the SID Phe+Tyr requirement estimate is approximately 92% of Lys. The diets with or without DAP or added Gly with a SID Lys:CP ratio of 7.0 in Exp. 1 would have contained a SID Phe+Tyr ratio of 90% of Lys. The Phe+Tyr ratio relative to Lys might be limiting, but because pigs responded in G:F with the addition of NPN from DAP or L-Gly, it is speculated that N for NEAA synthesis was more likely limiting.

The NRC (2012) suggests a SID Lys requirement of 1.23% for pigs weighing between 11 and 25 kg. In Exp. 1, all diets were ­formulated to a SID Lys level of 1.25%, which is similar to the NRC (2012) estimate for pigs in this weight range. However, the pigs would be below the requirement estimate of 11.1 g/d SID Lys based on feed intake, as the treatment with the highest ADFI (7.0% SID Lys:CP) would have only consumed 10.8 g/d SID Lys. Many studies have evaluated the optimal SID Lys level for pigs in this weight range. Kendall et al. (2008) conducted five experiments and ­estimated a requirement of 1.30% SID Lys for pigs weighing 11 to 27 kg. However, there are few studies evaluating the effect of SID Lys:CP in diets that are adequate or limiting in SID Lys. Therefore, Exp. 2 evaluated the effect of SID Lys both below (1.15%) and above (1.30%) the NRC requirement estimate to better determine the optimal SID Lys:CP ratio. Millet et al. (2018) evaluated the effects of varying dietary CP concentrations and two SID Lys levels (1.0 and 1.1%) in pigs weaned at 8.3 kg. They observed that ADG increased as the CP level increased, possibly due to the ­availability of N in the diets. Additionally, Millet et al. (2018) observed an interaction where at low CP levels, the performance differences between the two SID Lys levels were minimal, possibly because N was first limiting before Lys. At higher CP levels, pigs fed diets with increased SID Lys exhibited increased G:F. In Exp. 2, increasing SID Lys improved overall pig performance. Additionally, the observed SID Lys:CP × SID Lys interaction suggests that pigs fed 1.15% SID Lys were more responsive to increasing SID Lys:CP ratio than those fed 1.30% SID Lys, likely because they were further below their SID Lys requirement. This data suggests that when SID Lys is provided at an adequate level, the SID Lys:CP ratio becomes less critical compared to when both SID Lys and N are limiting.

Previous data suggests that high SBM levels in diets for 10 to 25 kg pigs can impair growth performance (Faccin et al. 2024). Thus, decreased performance at 6.01% SID Lys:CP in the corn-soy diet in Exp. 3 may be attributed to the high SBM content (37.8%). Therefore, the optimal SID Lys:CP ratio may be confounded with the SBM concentration within the diet. In Exp. 3, the SID Lys:CP × DDGS interaction for G:F indicates that the response to SID Lys:CP differed between the two DDGS levels, which may be driven by the high SBM content of the corn-SBM diet without DDGS at 6.01% SID Lys:CP. For diets containing 15% DDGS, G:F peaked at 6.47% SID Lys:CP, aligning with data from Exp. 1 and 2 and previous literature evaluating SID Lys:CP ratios (Millet et al. 2018; Rocha et al. 2022). In the corn-SBM diets without DDGS, maximum G:F was observed at a calculated SID Lys:CP level of 6.69%. It is important to note that these ratios are calculated values based on the analyzed composition of ingredients (SBM, corn, and DDGS) before the study. A potential explanation for the slightly higher estimate for SID Lys:CP could be that the CP content of diets from analyzed ingredients was slightly lower than CP of analyzed diets. Thus, the estimated SID Lys:CP ratios shift slightly. The corn-SBM diet formulated to 6.70% SID Lys:CP would have an approximate ­calculated ratio of 6.60% while the 6.45% SID Lys:CP diet with DDGS has a calculated ratio of approximately 6.52%. Nevertheless, main effects indicate that maximal G:F was observed at 6.45% SID Lys:CP, which is consistent with the previous literature (Millet et al. 2018; Rocha et al. 2022).

Instead of using total CP, formulating diets based on SID Lys:SID CP may serve as a strategy to ensure sufficient N availability for NEAA synthesis. It may also be a proxy for meeting limiting AA that do not have well-defined requirement estimates, such as His or Phe+Tyr. The NRC (2012) estimates a SID N requirement of 2.56% for 11- to 25-kg pigs, which corresponds to a calculated ratio of 7.69% SID Lys:SID CP. Expressing CP on a SID basis would improve precision by estimating digestible N available to the pig; however, this approach is not commonly used in industry practice. In Exp. 1, the moderate feed-grade AA diet had a SID Lys:SID CP ratio of 7.68%, beyond which G:F decreased. A similar trend was observed in Exp. 2, where growth performance decreased when the SID Lys:SID CP ratio exceeded 7.60%. In Exp. 3, maximum G:F was observed at a SID Lys:SID CP ratio of 7.88% in the 0% DDGS diets and 7.59% in the 15% DDGS diets. Either method supports the concept that a high SID Lys:CP or SID Lys:SID CP ratio may result in insufficient N for NEAA synthesis and impair growth performance.

In summary, these data suggest that a SID Lys:CP ratio of ­approximately 6.5% is optimal for pigs weighing 11- to 25-kg, aligning closely with the calculated NRC (2012) estimate. On an SID CP basis this equates to a SID Lys:SID CP ratio of approximately 7.7%. When the ratio exceeds this value, growth performance, particularly feed efficiency, is compromised likely because N from CP becomes limiting for the synthesis of NEAA. Feed efficiency can be recovered by incorporating a N source, such as NPN like DAP or amino N sources like Gly.

## Abbreviations:

AA: amino acid
ADG: average daily gain
ADFI: average daily feed intake
ATTD: apparent total tract digestibility
BUN: blood urea nitrogen
BW: body weight
CP: crude protein
DAP: diammonium phosphate
DDGS: distillers dried grains with solubles
DM: dry matter
EAA: essential amino acids
G:F: gain-to-feed ratio
N: nitrogen
NEAA: non-essential amino acids
NPN: non-protein nitrogen
SID Lys:CP: standardized ileal digestible lysine to crude protein ratio
